# Silicosis history: from antiquity to the anthropocene

**DOI:** 10.3389/fpubh.2026.1898046

**Published:** 2026-07-27

**Authors:** Serap Kurt Kayserili, Metin Akgün

**Affiliations:** 1 Department of Social Studies Education, Faculty of Education, Aǧri İbrahim Çeçen University, Aǧri, Türkiye; 2 Department of Pulmonary Diseases, School of Medicine, Aǧri İbrahim Çeçen University, Aǧri, Türkiye; 3 Department of Systematic Philosophy and Logic, Faculty of Literature, Atatürk University, Erzurum, Türkiye

**Keywords:** crystalline silica, denim sandblasting, engineered stone, health policy, history of medicine, occupational health equity, occupational lung disease, silicosis

## Abstract

Silicosis is the oldest occupational lung disease known to medicine and, at the same time, one of the most contemporary public health emergencies. Described in classical antiquity, given monographic attention by Paracelsus and Agricola, established as the founding example of occupational medicine by Ramazzini in 1700, and named by Visconti in 1870, the disease was widely assumed by the late 20th century to be a controlled relic of heavy industry. Yet over the past two decades it has returned with force as an acute and accelerated disease of the young, through two technology-driven outbreaks: denim sandblasting and the fabrication of engineered (artificial) stone. This article traces silicosis across roughly 2,500 years and argues that its history is best understood less as a story of mineralogy than as one of political economy: of who works, who profits, who is believed, and who is compensated. This article integrates three dimensions usually treated apart: the medical history of recognition and pathophysiology; the social history of class, race and the deliberate manufacture of doubt; and the history of labor mobilization and health policy, from the world's first silicosis compensation statute (South Africa, 1911) and the 1930 Johannesburg conference of the International Labor Office (ILO) to the United States silica standard of 2016 and Australia's world-first prohibition of engineered stone in 2024. As Sisyphus was condemned to roll a boulder uphill only to watch it roll back each time he neared the summit, so each silicosis epidemic that one society defeats tends to reappear in another; we argue that this cyclical recurrence is driven by economic disparity. Each time a wealthy jurisdiction eliminates a hazardous process, that process, and with it the disease, migrates to a poorer country or a less-regulated sector rather than disappearing, and until those underlying inequalities are addressed the boulder will keep rolling back down. This article closes with a forward projection on emerging hazards, diagnostics, the prospects for the World Health Organization-ILO goal of global elimination, and the equity question of who is left behind. Silicosis is wholly preventable; its persistence is therefore a political choice, not a scientific mystery.

## 1 Introduction

Few diagnoses compress the entire arc of occupational medicine into a single disease as completely as silicosis does. It can be traced, in descriptive form, to the breathlessness Hippocrates noted in metal diggers and stone-quarry workers in the fifth century before the common era; it was given monographic attention by Paracelsus and Agricola in the

16th century; it became the founding example of Bernardino Ramazzini's new discipline of occupational medicine in 1700; it acquired its modern name from the Italian pathologist Achille Visconti in 1870; and, in the early 20th century, it generated some of the first occupational disease compensation laws anywhere in the world. For much of the late 20th century, clinicians in high-income countries came to regard silicosis as a controlled and almost historical condition, a slow disease of old miners fading steadily under regulation and engineering control. This review argues that silicosis persists not because we lack the means to prevent it, but because of political and economic choices about whose health is protected.

That confidence was misplaced. Over the past two decades, silicosis has re-emerged as an acute and accelerated disease of the young: among denim sandblasters in their teens and twenties, and among engineered stone fabricators, many of them under thirty-five, who now require lung transplantation at ages once thought implausible for a pneumoconiosis. The oldest occupational lung disease is, once again, killing the youngest workers. That so ancient a disease should resurge through novel industrial materials and processes makes it, in a real sense, a disease of the Anthropocene—the proposed epoch in which human industrial activity has become the dominant force shaping both the planet and the exposures its workers face. The pattern is not confined to one country or one industry; it recurs across geographies and eras with a regularity that demands explanation rather than mere description.

This review takes that recurrence as its organizing problem and borrows a figure from Greek mythology to name it. Sisyphus was condemned to roll a boulder up a mountain only to watch it roll back down each time he neared the summit, an image of endlessly repeated, never-completed labor (1, 2). Silicosis epidemics behave in much the same way: each time a society appears to have conquered the disease, the boulder rolls back, and a new epidemic appears somewhere else. Our central thesis is that this is not bad luck or scientific ignorance but the predictable consequence of economic disparity. Silicosis has never been primarily a question of dust and mineralogy; it is a question of political economy. The same silica that scarred the lungs of Roman quarry slaves scars the lungs of contemporary countertop fabricators, but whether exposure becomes disease, whether disease is recognized, and whether recognition produces compensation and prevention are determined by labor relations, industrial interests, and the politics of knowledge. When a wealthy jurisdiction bans a hazardous process, the process does not vanish; it relocates to a poorer country or a less-regulated sector, carrying the disease with it. Until the underlying inequalities of wealth, power and regulation are addressed, the cycle will continue.

To develop that argument we deliberately braid together three histories that are usually told apart, the medical, the social and the labor policy, and then carry the synthesis forward into a projection of where the disease is heading. We write jointly from pulmonary medicine and from social history precisely because the disease cannot be understood from either vantage alone. Throughout, we treat silicosis as a tracer: a condition whose changing visibility reveals less about biology than about power, and whose persistence measures the limits not of medicine but of political will.

This is a narrative, interpretive review. We searched PubMed, Scopus and Google Scholar, alongside the historical and social-science literature, for English-language sources on the history, pathophysiology, epidemiology and regulation of silicosis, and supplemented these with foundational monographs, primary historical accounts, and legal and institutional documents. Sources were selected for their relevance to the review's integrative argument, and we flag explicitly where the historical evidence is uncertain or contested.

## 2 The long prehistory of a “new” disease: antiquity to early modernity

Dust-related lung disease is almost as old as organized labor itself. Silica exposure would have accompanied the earliest flint-knapping, mining, quarrying and stone-working, and silicotic change has been described in the preserved lung tissue of ancient human remains, although such palaeopathological claims rest on limited material and should be read as plausible rather than firmly established (3). Organized mining capable of generating heavy respirable dust is documented in China, India and along the Nile from the fourth and third millennia before the common era, and the monumental architecture of antiquity was quarried and dressed by laborers who would have inhaled silica in large quantities.

In the classical record, Hippocrates (c. 460–370 BCE) described a state of breathlessness among those who dug metals and cut stone, the earliest clinical reference of its kind. Pliny the Older adult later noted that miners tied bladders or loose membranes over their faces as rudimentary respirators, an implicit recognition that the air of the mine was itself harmful; Lucretius, in *De rerum natura* (circa 55 BCE), remarked on how quickly miners perished (3). These observations were descriptive rather than aetiological, and it is important to acknowledge that the ancient category of “consumption” (phthisis) conflated silicosis, tuberculosis and other wasting diseases of the chest. We therefore present the ancient evidence as a record of awareness that certain kinds of work destroyed the lungs, not as proof of a specific modern diagnosis. That awareness, however, is itself historically significant: from the beginning, dust disease was understood as a disease of particular occupations, of the people who did society's hardest and dirtiest work.

The early modern period brought the first sustained attention. Georgius Agricola, in *De Re Metallica* (1556), described the lung diseases of Central European miners, recommended ventilation and face coverings, and famously recorded mountain women widowed many times over by mining “consumption” (4). Paracelsus devoted an entire treatise, *On the Miners' Sickness and Other Diseases of Miners* (Von der Bergsucht, written in the 1530s and published in 1567), to the occupational diseases of miners and smelter workers; it is arguably the first book dedicated to an occupational disease, and the source of the enduring maxim that the dose makes the poison. The decisive synthesis came from Bernardino Ramazzini, whose *De Morbis Artificum Diatriba* (“Diseases of Workers,” 1700; expanded 1713) examined more than fifty trades, described the sand-like material found at autopsy in the lungs of stonecutters, and insisted that physicians add to the Hippocratic bedside questions one further query: “what is your occupation?” With that question Ramazzini founded occupational medicine as a discipline and reframed dust disease as a product of work, and therefore of social organization, rather than of fate or individual constitution (5).

## 3 Naming the disease: 19th century nosology and the Industrial Revolution

Before silicosis had a name, it traveled under the vernacular labels of the trades that produced it: “grinders' asthma” and “grinders' rot” among the Sheffield cutlery workers, “potters' rot” in the ceramics districts, “stonecutters' consumption,” “knife-grinders' phthisis,” the Cornish tin miners' “miners' phthisis,” and the Japanese miners' term “yoroke” (physical instability). The sheer proliferation of names reflected the absence of a unifying etiology and, with it, the absence of any basis for unified prevention or compensation: a disease without a single name is difficult to regulate as a single hazard.

The Industrial Revolution sharpened the problem dramatically. Power drills, pneumatic hammers, mechanized grinding wheels and, in the 20th century, abrasive blasting liberated far more respirable dust than hand tools ever had, compressing into a few years of intense exposure a dose that might previously have accumulated over a working lifetime. Mechanization did not create silica disease, but it industrialized it, turning a chronic background affliction into recognizable, clustered epidemics. The same intensification would later reappear, in concentrated form, in the sandblasting booth and the stone-fabrication shop.

The modern nosology crystallized in the second half of the 19th century. Friedrich Albert von Zenker coined the umbrella term *pneumonokoniosis* (“dust lung”) in 1866 and described quartz-worker fibrosis the following year. The specific term silicosis, from the Latin *silex* (“flint”), was introduced by Achille Visconti in 1870 on the basis of an autopsy at the Ospedale Maggiore in Milan, and was formally published by Carlo Luigi Rovida in 1871 (3). Naming mattered far beyond pathology. By carving silica-induced fibrosis out of the undifferentiated mass of “consumption,” the new term made the disease, for the first time, a discrete entity that could be counted, attributed to a specific exposure, and therefore in principle prevented and compensated. The history of silicosis as a public and political problem begins, in a real sense, with its name.

## 4 The 20th century: disasters, compensation, and the politics of definition

### 4.1 South Africa and the first compensation laws

The gold mines of the Witwatersrand made silicosis a matter of state. Faced with extraordinary rates of disease among miners drilling high-silica rock at depth, South Africa introduced the world's first silicosis-specific compensation statute, the Miners' Phthisis Act of 1911 (consolidated in 1925), together with the first systematic medical surveillance of an industrial workforce (6). This was, in narrow terms, a genuine advance: a state had accepted that a particular dust caused a particular disease and that those it sickened were owed redress. Yet the same system encoded racial inequity from the outset. At the international conference convened in 1930, it was recorded that roughly 85% of the approximately £15 million paid in compensation between 1911 and 1929 had gone to white miners, who constituted only about a tenth of the workforce, while the Black migrant majority, drawn from across southern Africa under the migrant labor system, received a small fraction (3, 6). The first machinery of recognition was thus also machinery of exclusion. As Elaine Katz documents in The White Death, her foundational social history of silicosis on the Witwatersrand, the early South African system combined genuine epidemiological sophistication with a structural indifference to the Black migrant majority whose labor it consumed (7). From its earliest institutional form, silicosis compensation tracked the lines of race and power, and the migrant ex-miners of Lesotho, Mozambique and the Eastern Cape would wait generations for even partial redress. That redress came only in our own decade: in the class action Nkala and Others v Harmony Gold Mining Company, the South Gauteng High Court certified a silicosis-and-tuberculosis class in 2016, the parties signed a settlement in May 2018, and the court approved it in July 2019. The fund, roughly five billion Rand (about US$350 million) administered through the Tshiamiso Trust, was set aside for miners employed since 1965, with up to some hundred thousand potential beneficiaries across southern Africa (8). A century separated the first compensation statute from this belated reckoning, and the gap is the measure of how unequally the disease and its remedies have been distributed.

### 4.2 The 1930 Johannesburg conference and the “truncation” of the disease

In August 1930 the International Labor Office (ILO), together with the Transvaal Chamber of Mines, convened the International Silicosis Conference in Johannesburg. The conference standardized the nosology and etiology of silicosis and prepared the ground for the 1934 ILO Silicosis Convention, which recognized the disease internationally as compensable (9). Historians of medicine, however, have argued that the conference also “truncated” the disease. Under the strong influence of physicians employed by or sympathetic to the mining industry, the working definition was narrowed to radiologically advanced silicosis, excluding early disease, mixed-dust disease, and silica-related harms that did not fit the favored radiographic picture. The narrower the compensable definition, the smaller the employers' liability. This narrow definition is an early, well-documented and consequential example of a recurring phenomenon: the boundaries of a diagnosis can be drawn to serve interests as much as patients, and the apparatus of objective science can be enlisted to limit, rather than extend, the recognition of harm (3, 6).

### 4.3 Hawk's nest: race, class, and the worst industrial disaster in United States history

Between 1930 and 1931, workers driving the Hawk's Nest Tunnel near Gauley Bridge, West Virginia, bored through rock extraordinarily rich in silica to build a hydroelectric water-diversion tunnel. They frequently dry-drilled with minimal protection; contemporaneous accounts indicate that wet methods, which suppress dust, were used chiefly when inspectors were expected. The workforce was largely composed of African-American migrant laborers, and the dead, whose numbers are variously estimated at the company's admitted 109, a congressional figure of 476 for 1930–35, and a memorial count of roughly 764 (with some historians proposing higher totals), could not in many cases be buried in white cemeteries. We present the death toll deliberately as a contested range rather than a single number, because the uncertainty is itself part of the history: the victims were poor, Black and migratory, and were therefore not fully counted in life or in death. The disaster, and the 1936 congressional hearing that followed, forced silicosis onto American front pages, into the dust-suppression campaigns of Frances Perkins's Department of Labor, and into the national consciousness as a social crisis; Martin Cherniack's history aptly subtitles it America's worst industrial disaster (10). It entered American cultural memory as well: in 1938 the poet Muriel Rukeyser, who had traveled to Gauley Bridge to report on the congressional hearing, published the documentary poem cycle The Book of the Dead, weaving trial testimony, medical evidence and the voices of the dying into one of the period's most powerful indictments of industrial negligence (11). Critics have noted that even this sympathetic account tended to subordinate the specifically racial dimension of the catastrophe, a reminder that the erasure of Black suffering operated in the documentary record as well as in the cemeteries. Hawk's Nest remains the paradigmatic case of how class and race determine not only who is exposed to silica but whose suffering is counted, recorded and remembered.

### 4.4 Classification, carcinogenicity, and exposure limits

The 20th century also produced the technical apparatus of control. The ILO's International Classification of Radiographs of Pneumoconioses, prefigured at the 1930 conference and first issued in 1950, standardized the reading of chest radiographs, grading the profusion of small opacities and the categories of large opacities that mark progressive massive fibrosis, and remains in use through successive revisions, including a fully digital edition in 2022 (12). In 1997 the International Agency for Research on Cancer reclassified crystalline silica from a probable to a confirmed Group 1 human carcinogen (13). Regulatory exposure limits tightened over decades, culminating in the United States Occupational Safety and Health Administration's landmark 2016 standard, which set a permissible exposure limit of 50 μg/m^3^ averaged over 8 h, with an action level of 25 μg/m^3^ (14); the American Conference of Governmental Industrial Hygienists threshold limit value stands at 25 μg/m^3^ (15), below the United States National Institute for Occupational Safety and Health recommended exposure limit of 50 μg/m^3^ (16); many occupational-health authorities regard this lower value as the minimum defensible target. The existence of this apparatus is essential to the argument that follows: silicosis is not a disease we lack the tools to prevent. We possess the classification, the carcinogenicity determination, the exposure science and the engineering controls. What is repeatedly lacking is the will, and the economic incentive, to apply them everywhere rather than only where workers can demand it.

## 5 What the dust does: pathophysiology in brief

**Figure 1:**
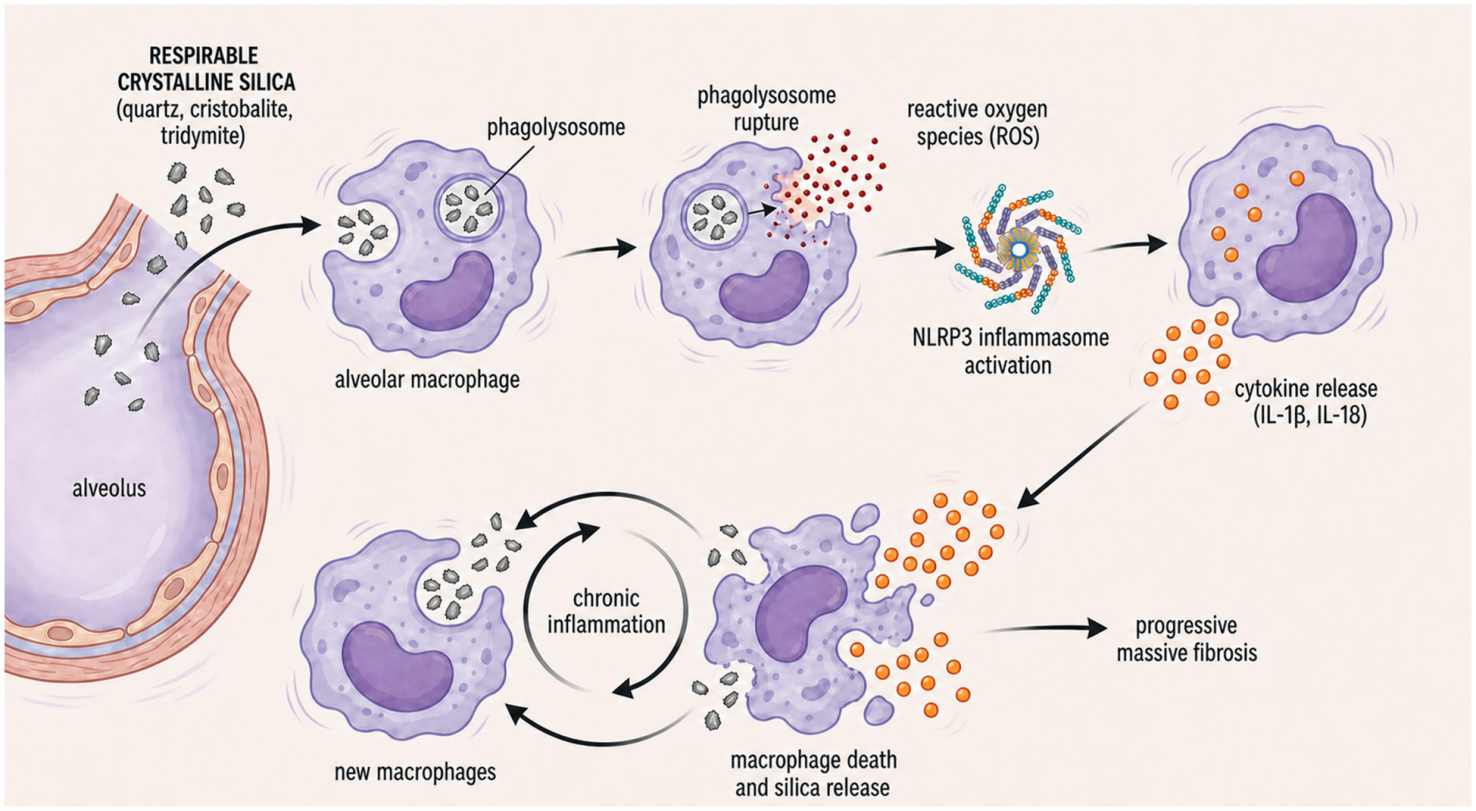
Cellular mechanism of silicosis. Inhaled respirable crystalline silica (quartz, cristobalite, tridymite) is phagocytosed by alveolar macrophages; phagolysosome rupture and reactive oxygen species (ROS) activate the NLRP3 inflammasome, driving release of interleukin-1β (IL-1β) and interleukin-18 (IL-18). The macrophage dies and liberates its indestructible silica, which is re-ingested by new macrophages, sustaining self-perpetuating inflammation that leads, through coalescing silicotic nodules, to progressive massive fibrosis (Illustration: AI-generated by the authors).

The mechanistic understanding of silicosis now reaches to the molecular level, and it explains both why the disease is self-perpetuating and why, in its acute forms, it can be so devastatingly rapid. Respirable crystalline silica, principally quartz, together with the more pathogenic cristobalite and tridymite generated when silica is heated (as in the curing of engineered stone resins), is inhaled deep into the alveoli and phagocytosed by alveolar macrophages. Rupture of the phagolysosome and the generation of reactive oxygen species activate the NLRP3 inflammasome (an intracellular protein complex that acts as primary sensor for cellular stress and damage), driving the maturation and release of the cytokines interleukin-1β and interleukin-18 and initiating a cycle of non-resolving inflammation (17). The macrophage dies, liberating its silica burden to be re-ingested by other macrophages, so that a single dose of indestructible mineral sustains inflammation indefinitely. The result is the characteristic silicotic nodule, a whorl of collagen, and, on coalescence of nodules, progressive massive fibrosis (Figure 1).

Clinically the disease presents in three forms (Table 1), each progressing at a pace that reflects the intensity of exposure (18). Chronic silicosis follows a decade or more of moderate exposure. Accelerated silicosis appears within roughly five to ten years of heavy exposure. Acute silicosis, or silicoproteinosis, develops within weeks to a few years of overwhelming exposure. This is the form that killed the Hawk's Nest workers and that now afflicts the youngest denim and engineered stone workers, in whom the freshly fractured, ultrafine, high-concentration dust of the booth and the fabrication shop reproduces, in a modern setting, the most extreme exposures of the industrial past. Critically, the disease may progress even after exposure ceases: longitudinal follow-up of former denim sandblasters has documented radiographic progression in 96% of those monitored at four years and in effectively all of them at ten years, despite the cessation of work (19, 20). Silica exposure is additionally associated with pulmonary tuberculosis, chronic obstructive pulmonary disease, lung cancer, chronic kidney disease and autoimmune conditions (13, 18). These mechanisms matter for the argument of this review in one decisive respect: because the disease is driven by an indestructible mineral and a self-sustaining inflammatory loop, and because it progresses after exposure ends, there is no safe way to “manage” ongoing exposure. The only effective intervention is to prevent the dust from being inhaled in the first place.

**Table 1:** Clinical forms of silicosis by exposure intensity and latency.

Form	Exposure intensity	Typical latency/onset	Note
Chronic silicosis	Moderate	≥ 10 years	Classic, slowly progressive
Accelerated silicosis	Heavy	~ 5–10 years	Faster progression
Acute silicosis (silicoproteinosis)	Overwhelming	Weeks to a few years	Seen in denim sandblasters and engineered stone workers; often fatal

## 6 The contemporary transformation: old dust, new industries

### 6.1 Denim sandblasting and the Turkish epidemic

**Figure 2:**
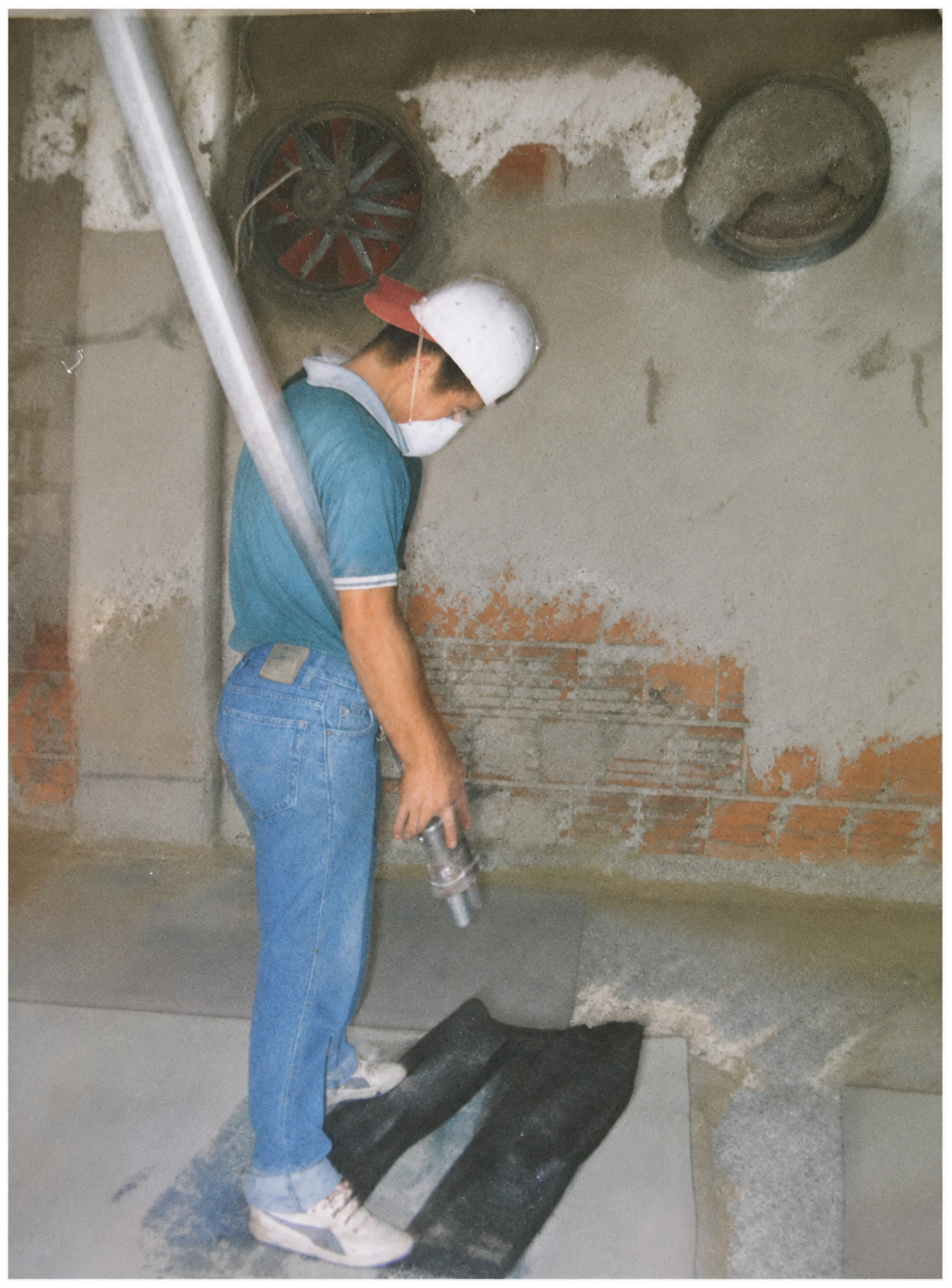
A young denim sandblaster at work in a small, enclosed, poorly ventilated workshop in Türkiye, directing a jet of silica sand onto a pair of jeans to produce a faded, “distressed” finish. In such an enclosed space the airborne silica is trapped and concentrated; personal protection is limited to a single disposable dust mask, and the only ventilation is the wall-mounted fan behind him. Reproduced with the kind permission of Abdulhalim Demir—silicosis patient, labor-rights activist, and founder of the Clean Clothes Campaign Türkiye.

Sandblasting raw denim with silica sand to give jeans a worn, faded, fashionably distressed appearance is a paradigmatic example of a hazard migrating along the path of least regulation. After the abrasive blasting of garments was restricted in the United Kingdom and then across much of Europe in the mid-twentieth century, the practice took root in Türkiye, frequently in small, poorly ventilated workshops employing very young men, some of whom began the work as adolescents (Figure 2), many unregistered and without social security (21, 22). The hazard announced itself when two young denim sandblasters received diagnoses of silicosis in close succession; the cases were presented internationally at the European Respiratory Society Congress in Copenhagen in 2005 and reported as concomitant cases the same year (23). The complexity of the occupation initially complicated diagnosis, and the severity of the problem remained underrecognised within Türkiye until a national television broadcast in 2007 catalyzed public awareness and prompted significant labor shifts as workers grasped the implications of the work (21).

**Figure 3:**
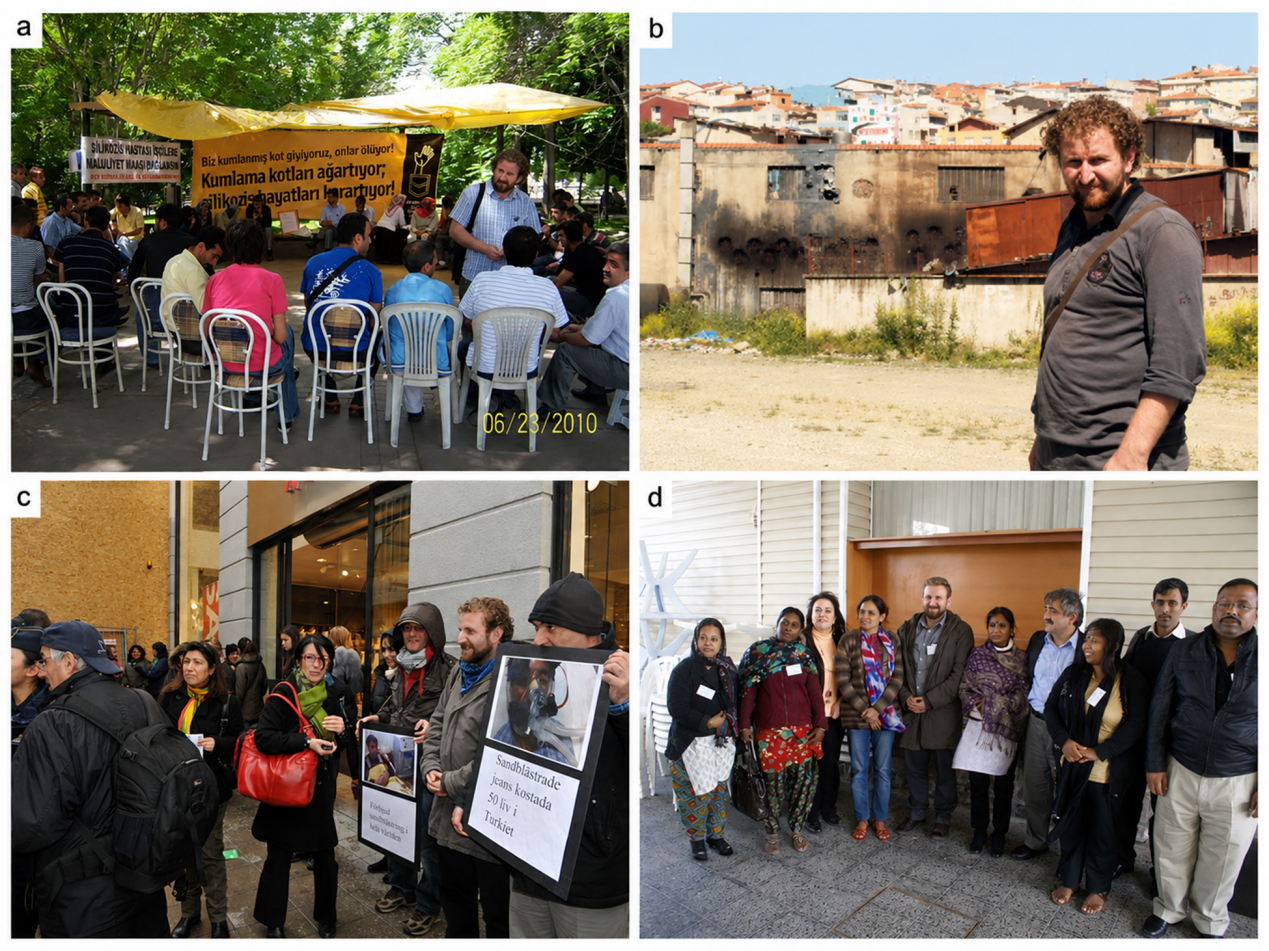
Worker mobilization and civil-society campaigning around denim sandblasting, in Türkiye and abroad. **(a)** Former sandblasters demonstrate for their rights at Güven Park, Ankara (2010). **(b)** A workshop that turned to chemical processing after sandblasting, where chemical attack has eroded the wall. **(c)** A 2010 street action in Stockholm urging clothing brands to ban sandblasting across their supply chains; a major retailer did so the same year. **(d)** Campaigners with Bangladeshi trade-union and civil society representatives at a 2010 forum, where efforts began to prevent the hazard's migration to Bangladesh. Reproduced with the kind permission of Abdulhalim Demir—silicosis patient, labor-rights activist, and founder of the Clean Clothes Campaign Türkiye.

The defining epidemiological account, published in 2008, comprehensively illuminated the conditions in these workplaces. It identified underage labor, unregistered employment and the absence of social security; it showed that workers who slept on site effectively doubled their exposure; and it found that 53% of former sandblasters had developed silicosis (24, 25). Türkiye prohibited the use of silica in sandblasting in 2009, a genuine milestone in occupational health, won in significant part through the mobilization of affected workers, civil society campaigning and international attention (Figure 3). Brazil, another middle-income country, had in fact banned sand abrasive blasting across all industries four years earlier, in 2005 (26), showing that such prevention is achievable in lower-income settings. Yet, as has been argued at length, the prohibition did not eliminate the practice; it relocated it to regions with less stringent regulation, notably Bangladesh, exporting the hazard rather than abolishing it (21). Worse, even within Türkiye the implementation of the ILO radiographic classification proved insufficient even in registered workplaces, compromising the protection and compensation the system was meant to provide (27). Subsequent follow-up has been sobering: disease progression reached 96% at four years and 100% at ten years among those monitored even after exposure ceased, and former sandblasters, despite an average exposure of only about three years, die at a significantly younger median age than the general population of their regions (19, 20, 28). The denim epidemic is thus at once a success story and a cautionary tale: prohibition worked locally and failed globally, because the economic conditions that created the demand for cheap distressed denim were never addressed. The distressed look itself, however, can now be achieved without silica—through laser abrasion, automated finishing systems, ozone treatment and enzymatic washing—so that sandblasting persists by reason of cost, not technical necessity (29).

### 6.2 Engineered (artificial) stone: the global frontier

If denim sandblasting showed how a hazard migrates between countries, engineered stone shows how it migrates between sectors. Engineered stone, used predominantly for kitchen and bathroom countertops, commonly contains more than 90% crystalline silica bound in a polymer resin, far exceeding the silica content of most natural granite or marble (30). Cutting, grinding and polishing it releases extreme concentrations of fine, often freshly fractured, and partly resin-bound respirable silica. Beyond crystalline silica, the same processes can liberate other inhalable hazards—volatile organic compounds such as styrene, benzene and toluene, and metals such as cobalt, aluminum, titanium and titanium dioxide derived from the resins, pigments, curing agents, accelerators and colorants—so that fabricators face a complex mixture of exposures rather than crystalline silica alone (30–32). The defining clinical series, reported from Israel in 2012, documented severe disease among fabricators, a large proportion of whom became candidates for lung transplantation (33). Comparable clusters were soon described in Spain, Italy, Australia, the United States and elsewhere, characteristically in workers under thirty-five, many of them, as in the United States, immigrants, and the disease can progress rapidly even after exposure ceases (34).

The policy response in the most affected high-income jurisdictions has been the most decisive in the history of the disease. Australia became the first country in the world to prohibit the manufacture, supply, processing and installation of engineered stone containing 1% or more crystalline silica; work-health-and-safety ministers agreed unanimously in December 2023, the ban took effect on 1 July 2024, and an import prohibition followed (35). Australian regulators concluded explicitly that no safe threshold of crystalline silica content in engineered stone had been demonstrated and that products with lower silica concentrations could not be assumed to be safe. In the United States, where federal and state authorities have described “deadly countertops” and an urgent need to eliminate the disease among stone workers, California, currently the principal jurisdiction systematically tracking the condition, has reported rapidly rising case counts, deaths among workers in their thirties and forties, and dozens of lung transplants, with the large majority of cases confirmed within only the most recent few years and model-based projections of many hundreds more (36). Australian trade unions have characterized engineered stone as “the asbestos of the 2020s” (37)—a comparison echoed in the occupational health literature, which frames the outbreak as a preventable repetition of past failures to control a lethal dust hazard (36). The crucial point for our argument is that the engineered stone epidemic arose after the denim epidemic was already well documented: the lesson of one high-silica, high-intensity process was available but not applied to the next. The boulder rolled back.

### 6.3 Global burden, the coal parallel, and the limits of counting

Global estimates capture only part of this picture. The Global Burden of Disease study attributed approximately 13,000 deaths and several hundred thousand disability-adjusted life-years to silicosis worldwide in 2019. The overwhelming majority of the silicosis burden occurred in men; the absolute number of incident cases has risen substantially since 1990, even as age-standardized rates declined. Because such estimates rely on coded mortality and on registered cases, they almost certainly undercount disease in informal, artisanal and unregistered sectors, precisely the populations to which hazardous processes are displaced when they are banned elsewhere. The true global burden is therefore very likely higher than the headline figures suggest, and is concentrated where it is least well measured (38, 39). A closely related warning sign comes from the United States coalfields, where progressive massive fibrosis has resurged among central Appalachian miners, now appearing in miners under the age of forty and attributed in significant part to the cutting of high-silica rock as coal seams thin (40). A disease officially classed as coal workers' pneumoconiosis is, at its mechanistic core, a silica story: a further illustration of how the same hazard reappears under different sectoral names whenever the economics of extraction push workers into siliceous rock.

## 7 The connective tissue: manufactured doubt, race, and the economics of displacement

Read together, the episodes above reveal a single recurring pattern with three elements: the deliberate manufacture of doubt, the tracking of the disease along the fault lines of race and class, and, binding the other two, the economics of displacement. Each element is well attested in the scholarly literature, and together they explain why a wholly preventable but incurable disease has proven so stubbornly recurrent.

### 7.1 Manufactured doubt and the production of ignorance

The first element is the deliberate production of scientific uncertainty. The pattern is older than the term for it. It begins, in the silicosis story, with the narrowing of the compensable definition at the Johannesburg conference in 1930 under the influence of industry physicians, and it runs through the mid-century activities of employer-funded research bodies. David Rosner and Gerald Markowitz, in *Deadly Dust*, traced how the American silica industry and its allies worked to shape what counted as silicosis, how it was measured, and how readily it was attributed to work; in their later *Deceit and Denial* they generalized the argument to industrial pollution more broadly (41, 42). Scholars of what Robert Proctor and Londa Schiebinger call agnotology, the study of culturally produced ignorance, have shown that uncertainty is often not a passive gap in knowledge but an actively manufactured product, and the former United States Assistant Secretary of Labor David Michaels, in *Doubt Is Their Product*, documented how the very techniques pioneered around occupational hazards such as silica were subsequently deployed across one industry after another. The contemporary form is familiar: the engineered stone industry's position that its product is safe “if handled correctly” and that liability rests with downstream fabrication shops, rather than with a material that is more than 90% crystalline silica, reproduces almost exactly the rhetorical strategy that the mining and foundry industries deployed a century earlier. The international historiography assembled in Paul-André Rosental's *Silicosis: A World History* makes clear that this is not a national peculiarity but a feature of how the disease has been governed across very different political and economic systems (3, 43, 44).

### 7.2 Race, class, and the unequal distribution of recognition

The second element is the persistent concentration of the disease, and of the failure to recognize it, along the fault lines of race and class. This is not incidental to the history of silicosis but constitutive of it. The Black migrant dead of Hawk's Nest, who could not be buried in white cemeteries and whose numbers remain uncertain precisely because they were not counted; the South African compensation system that directed the overwhelming majority of its payments to white miners while the Black migrant majority who did the most dangerous underground work received a fraction, and whose descendants secured meaningful redress only with the Nkala settlement of 2018–2019; and the predominantly immigrant, often undocumented Latino fabricators who dominate the engineered stone workforce in California today are not separate stories but a single story told repeatedly across a century and three continents. Alan Derickson's history of the Western miners' struggle shows that where workers organized and built their own health institutions they could force recognition, and that where they could not, the disease remained invisible. The lesson is consistent: silica disease concentrates wherever workers have the least power to refuse dangerous work, to demand its remediation, or to compel the recording of their own deaths (45). Recognition is not distributed according to suffering; it is distributed according to power.

### 7.3 The economics of displacement

The third element, which binds the other two, is the economics of displacement, and it is here that the Sisyphus metaphor becomes analytic rather than merely literary. Each time a wealthy jurisdiction eliminates a hazardous process, Britain restricting the abrasive blasting of garments in the mid-twentieth century, Türkiye banning denim sandblasting in 2009, Australia prohibiting engineered stone in 2024, the underlying consumer demand for the cheap product does not disappear, and neither does the supply of cheap, lightly regulated labor willing or compelled to produce it. The hazardous process therefore migrates rather than vanishing: from rich country to poor country, from the registered factory to the informal workshop, from one sector to another. Prohibition in one place becomes exposure in another. Türkiye's ban was followed by sandblasting in Bangladesh, and Australia's by the prospect of fabrication wherever engineered stone, or a similar high-silica material, is next worked without controls. The migration is driven by price differentials and regulatory gradients, which is to say by inequality itself. So long as there are large disparities in wealth, regulatory capacity and worker power between and within countries, the elimination of silicosis in one location will tend to reproduce it in another. The disease is, in this precise sense, a disease of inequality, a point that the occupational health community has increasingly framed in terms of global health equity and the question of who is left behind. It follows that silicosis will not be eliminated by better respirators or stricter local rules alone, but only by addressing the economic gradient that continually relocates the hazard to wherever workers are cheapest and least protected: through enforceable international standards, supply chain and brand accountability, and the deliberate reshaping of consumer demand away from products whose low price is paid for in the health of distant workers.

Two mechanisms should be distinguished. The first is displacement between countries, as a hazardous process banned in a richer state reappears in a poorer one. The second is displacement between sectors: engineered stone silicosis emerged inside wealthy countries, among immigrant and otherwise marginalized fabricators, falling on the least protected workers within a rich economy rather than crossing a border. Nor is political economy the sole driver: scientific lag around new materials, regulatory delay, and fashion and consumer cycles all contribute. Our claim is that these proximate factors operate within, and are repeatedly subordinated to, an underlying gradient of wealth, power and regulation.

## 8 Forward projection: where silicosis is heading

### 8.1 Emerging hazards

The lesson of two and a half millennia is that every technology which liberates fresh, fine silica tends to generate a new epidemic before regulation catches up. The next exposures are already visible: high-silica rock cutting in mechanized coal and metal mining; the vast, rapidly growing and largely informal world of artisanal and small-scale mining across low- and middle-income countries; tunneling and the handling of hydraulic-fracturing sand; novel composite and manufactured materials; and the “low-silica” engineered stone substitutes now being marketed, the long-term safety of which remains unproven and must not be assumed without evidence. If the displacement dynamic holds, the highest future burden will fall not where engineered stone has been banned but where its fabrication, or some functionally similar high-silica process, is taken up next under weak regulation.

### 8.2 Diagnosis and management

Diagnostic capacity is improving. High-resolution and low-dose computed tomography outperform plain radiography in detecting early disease; the ILO classification now exists in a digital edition; image reading assisted by artificial intelligence is developing rapidly; and physiological tools such as impulse oscillometry offer the prospect of detecting early functional change in young, asymptomatic but exposed workers, when intervention by removal from exposure can still alter the trajectory. Exploratory biomarkers, including inflammasome-related cytokines and serum markers of epithelial and macrophage injury, may eventually permit earlier and more specific identification of those at risk. No curative therapy exists; management remains supportive, with lung transplantation reserved for end-stage disease. The NLRP3-interleukin-1 axis and the antifibrotic agents now used in other progressive fibrotic lung diseases are biologically plausible future targets and should be framed honestly as research directions rather than established treatments. Diagnostic and therapeutic advances are welcome, but they address the consequences of exposure; on their own they cannot break the Sisyphean cycle, because that cycle is generated upstream, by the social and economic conditions that put workers in front of the dust.

### 8.3 Elimination: prospect and obstacle

The International Labor Office and the World Health Organization launched a Global Programme for the Elimination of Silicosis in 1995, with the aspiration of eliminating the disease as a public health problem by 2030, supported by national programmes and regional initiatives such as the elimination effort in the Americas (46). This target will not be met. The reasons are not scientific but political and economic: informal labor beyond the reach of inspection, weak or absent enforcement, the cross-border migration of hazardous processes, and the persistence of manufactured doubt. An honest projection must therefore be sober. Yet the same history that documents repeated failure also documents that prevention works when it is genuinely willed and adequately resourced: Britain's early restriction of abrasive blasting, the denim ban in Brazil in 2005 and in Türkiye in 2009, within their own borders, and Australia's 2024 engineered stone prohibition all show that, locally, the disease yields to policy. The unfinished task is to make prevention global and to couple it with the reduction of the economic disparities that drive displacement, through enforceable international standards, supply chain and brand accountability, consumer awareness campaigns that reshape demand toward safer products, and support for regulatory capacity in the countries to which hazardous work is currently exported.

### 8.4 Controversies, research gaps, and competing schools of thought

Several genuine controversies and unresolved questions structure the field and define its research frontier. The first is the safe threshold debate: regulatory authorities and industry have long argued over whether any exposure limit is truly protective, with occupational hygiene bodies progressively lowering permissible limits while some authorities, and the Australian engineered stone decision, conclude that for products with a high concentration of silica no safe threshold has been demonstrated. The second is the question of attribution and definition that has divided the field since the 1930 Johannesburg conference: how much early or mixed-dust disease should count as compensable silicosis, and whether radiographic classification, designed for chronic disease, adequately captures the accelerated and acute forms now seen in young workers. A third, more recent dispute concerns whether “low-silica” engineered stone substitutes are meaningfully safer or merely a means of preserving a hazardous industry; the evidence base is currently insufficient to resolve it. Beyond these controversies, the principal research gaps are clear: the true global burden is poorly quantified because surveillance misses informal and artisanal workers; there are no validated, widely deployed early biomarkers that distinguish workers who will experience disease progression from those who will not; the natural history of engineered stone and denim-related disease after exposure ceases is still being characterized; no disease-modifying therapy has been established, leaving transplantation as the only option in end-stage disease; and the effectiveness of different policy instruments—bans, exposure limits, surveillance, supply chain accountability—has rarely been compared head to head. Closing these gaps will require linkage of clinical, epidemiological and policy research that the historically siloed study of silicosis has seldom achieved.

## 9 Discussion

**Figure 4:**
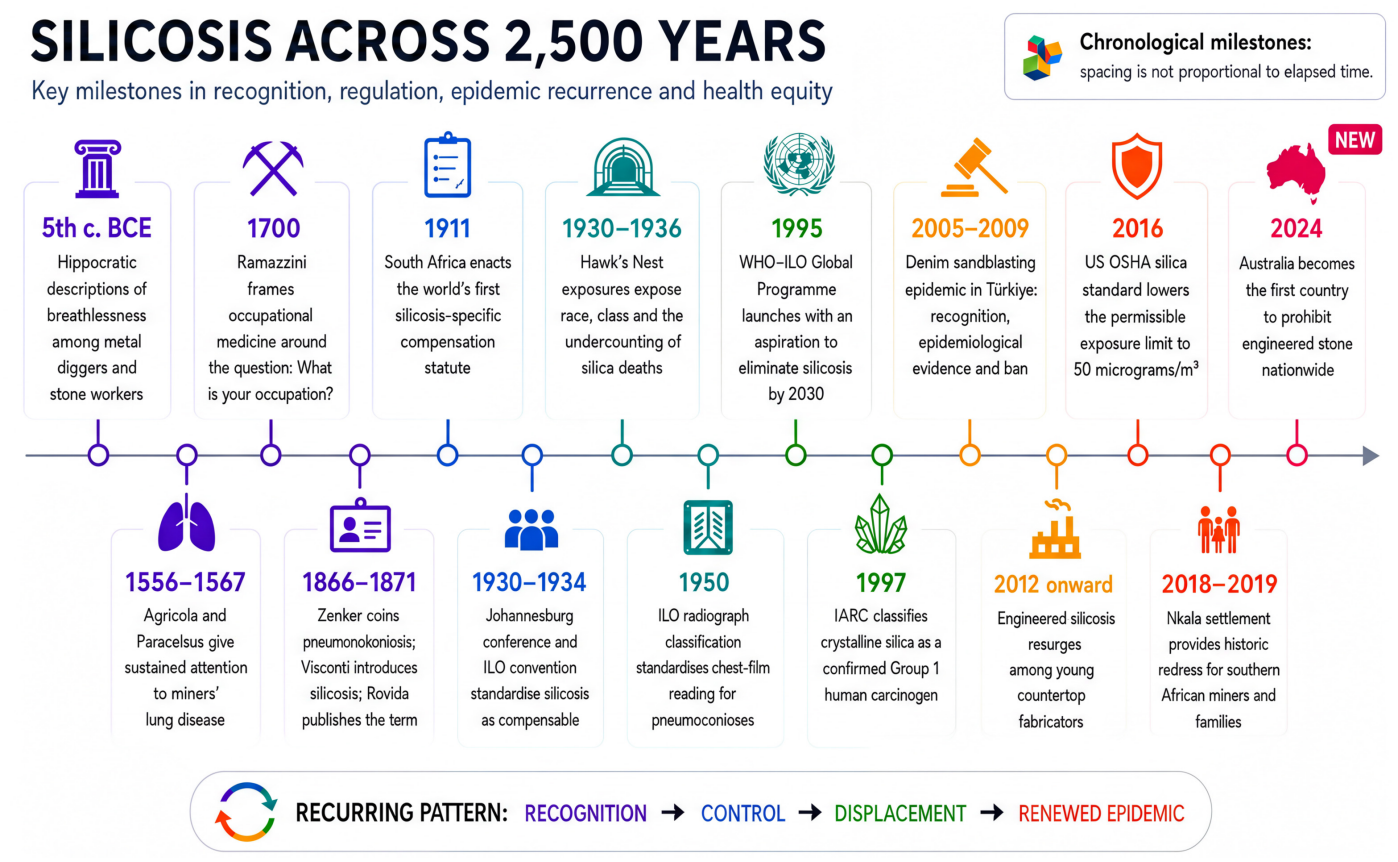
Selected milestones in silicosis across roughly 2,500 years—from Hippocrates' description of breathlessness in miners and stone workers to Australia's 2024 engineered stone ban. Milestones are chronological but not spaced proportionally to elapsed time, and together trace a recurring pattern of recognition → control → displacement → renewed epidemic (Illustration: AI-generated by the authors).

Drawing the medical, social and policy threads together, the central finding of this review is that silicosis is governed less by mineralogy than by political economy, and that its recurrence is therefore patterned and predictable rather than accidental. The myth of Sisyphus captures that pattern precisely (Figure 4).

In Albert Camus's reading of the myth, Sisyphus is the absurd hero who finds meaning in the very struggle that can never be completed (2). The analogy to silicosis is exact in its description and, we argue, escapable in its prognosis. We borrow Camus's image of endlessly repeated labor, but not its resignation: unlike his metaphysical absurd, the recurrence of silicosis is historical and contingent, and the boulder can be made to stay at the summit. The disease has recurred across geographies and eras with the regularity of the rolling boulder, and the lessons of past tragedies have not been fully integrated: the denim epidemic was thoroughly documented before the engineered stone epidemic gathered force, yet the second was not prevented by knowledge of the first. Reactive measures, diagnosing, compensating and, increasingly, transplanting, have consistently proven less effective and more costly, in lives and in money, than proactive prevention.

But silicosis, unlike Sisyphus's punishment, is not a predetermined fate (1). It is a preventable disease with a known cause, known controls and, in the case of engineered stone and denim sandblasting, the demonstrated option of eliminating the hazardous process altogether. The boulder keeps rolling back not because gravity is inescapable but because we keep rebuilding the hill, because the global economy continually recreates the conditions under which cheap, lightly regulated labor is set to work on materials with high silica contents. To alter Sisyphus's fate is therefore to change those conditions: to move away from production models that generate silicosis, to hold global supply chains and consumer demand to account, and to close, rather than exploit, the disparities in wealth and regulatory power that relocate the hazard from one vulnerable population to the next. Doing so requires a concerted global effort that values human lives over short-term economic gain. The choice of whether the boulder reaches the summit and stays there is, finally, ours.

Paradoxically, silicosis is the oldest occupational lung disease and, in its newest forms, one of the most modern industrial epidemics. The continuity across 2,500 years is not the dust alone but the social and economic relations that decide whose lungs receive it, whose disease is recognized, and whose suffering is recorded and redressed. The Sisyphean recurrence of silicosis epidemics, from the gold mines of the Witwatersrand in South Africa to coal mining in Gauley Bridge in the United States, and onward to wherever the hazard is next displaced, will continue until the economic disparities that drive that displacement are themselves addressed. Written jointly from pulmonary medicine and social history, this review has sought to make that pattern, and that choice, visible. The history of silicosis is, in the end, a history of justice repeatedly deferred; whether it stays that way is a matter of choice rather than necessity.
